# Osteoprotective effect of combination therapy of low-dose oestradiol with G15, a specific antagonist of GPR30/GPER in ovariectomy-induced osteoporotic rats

**DOI:** 10.1042/BSR20150146

**Published:** 2015-08-18

**Authors:** Wen-Bo Kang, Yu Cong, Jiang-Ying Ru, Si-Qi Ying, Ting Zhu, Dong-Sheng Wang, Xiao-Wei Liu, Gang Liu, Jian-Ning Zhao

**Affiliations:** * Department of Orthopedics, Clinical School of Nanjing, Second Military Medical University, Nanjing 210002, China; † Department of Orthopedics, Jinling Hospital, School of Medicine, Nanjing University, Nanjing 210002, China; ‡ Department of Orthopedics, Yangzhou No 1 People's Hospital, Second Clinical School of Yangzhou University, Yangzhou 225000, China; § Department of Stomatology, Chongqing Medical University, Chongqing 400016, China; ∥ Department of Ultrasound, Xijing Hospital, Fourth Military Medical University, Xi'an 710032, China

**Keywords:** bone, G15, G protein-coupled oestrogen receptor 30 (GPR30/GPER), oestradiol (E2), osteoporosis, ovariectomy (OVX)

## Abstract

Identified and cloned in 1996 for the first time, G protein-coupled oestrogen receptor (ER) 30 (GPR30/GPER) has been a hot spot in the field of sex hormone research till now. In the present study, we examined the effects of low-dose oestradiol (E2) combined with G15, a specific antagonist of GPR30 on ovariectomy (OVX)-induced osteoporosis in rats. Female Sprague–Dawley (SD) rats undergoing OVX were used to evaluate the osteoprotective effect of the drugs. Administration of E2 [35 μg/kg, intraperitoneally (ip), three times/week) combining G15 (160 μg/kg, ip, three times/week) for 6 weeks was found to have prevented OVX-induced effects, including increase in bone turnover rate, decrease in bone mineral content (BMC) and bone mineral density (BMD), damage of bone structure and the aggravation in biomechanical properties of bone. The therapeutic effect of these two drugs in combination was better than that of E2 alone. Meanwhile, the administration of G15 prevented body weight increase or endometrium proliferation in the rats. In conclusion, administration of low-dose E2 combining G15 had a satisfactory bone protective effect for OVX rats, without significant influence on body weight or the uterus. This combination therapy may be an effective supplement of drugs in prevention and treatment for postmenopausal osteoporosis.

## Abstract (precis)

Administration of low-dose oestradiol (E2) combining G15, this combination therapy may be an effective supplement of drugs in prevention and treatment for postmenopausal osteoporosis.

## INTRODUCTION

Oestrogen plays critical roles in the metabolism of bone. The absorption and turnover of bone would be remarkably elevated in postmenopausal women when the level of oestradiol (E2) descends expeditiously, which could further lead to lower bone mass and higher fracture risk and progress to primary osteoporosis.

To be effective, it is necessary for oestrogen to bind to its receptors. Till now, there are three oestrogen receptors (ERs) identified, namely ERα [1], ERβ [2] and G protein-coupled ER 30 (GPR30/GPER) [3]. They have wide distributions in the body, especially the musculoskeletal system and the reproductive system. When patients with osteoporosis are treated with exogenous oestrogen for a long time, side effects such as endometrial hyperplasia, uterus and breast tumour would occur [4].

Therefore, the use of the unique characteristics of different ERs to improve the therapeutic effects and reduce the side effects in oestrogen replacement therapy (ERT) has been a vital issue remained to be solved.

Previous studies have shown that GPR30 may have close relationship with the metabolism of bone in rodent models [5,6]; however, those focusing mainly on GPR30 gene knockout (GPR30-KO) animals may not represent the wild-type (WT) ones well. So, in our study, we used low-dose E2 combined with G15 (a specific antagonist of GPR30) on WT Sprague–Dawley (SD) ovariectomy (OVX) rat models to discover the relationship between GPR30 and bone metabolism.

## MATERIALS AND METHODS

A total of 32 SPF (specefic pathogen free) 3-month-old nulliparous female SD rats weighted (230±20 g) were purchased from the Experimental Animal Center of the Fourth Military Medical University. After a 7-day adaptation period in open animal feeding room with temperature of (23±2°C), humidity of (60±5%), natural light, standard nutrition pellets (Ca 0.9%, P 0.7%) and clean water, the rats were randomly divided into two groups: Eight were anaesthetized by pentobarbital sodium [Bioszune, 40 mg/kg, intraperitoneally (ip)] firstly before sham operation and then treated with vehicle (BETIS olive oil, Torres Y Ribelles, 1.5 ml/kg, ip, three times/week) to represent the sham group; The other 24 underwent bilateral OVX [7] and were randomly divided into three groups with eight per group. Four weeks after surgery, the 24 rats were treated with either vehicle (eight rats treated with olive oil, 1.5 ml/kg, ip, three times/week were labelled as the ‘OVX’ model group) or E2 (eight rats treated with E2 benzoate injection 35 μg/kg, ip, three times/week, purchased from Hangzhou Animal Medicine Factory, were labelled as the ‘E2’ group). The other eight rats were treated with E2 plus G15 (Cayman, 160 μg/kg, ip, three times/week) and were labelled the ‘E2 + G15’ group for 6 weeks. Body weight of the rats was documented weekly during the experimental period. At the end of the sixth week, rats were anaesthetized and blood were then obtained from the inferior vena cava; the uteruses were removed and immediately weighed; the femurs were dissected and stored in 40% formaldehyde solution in room temperature until examined for structural analysis and biomechanical testing. All animals were treated according to the principles and procedures of the NIH Guide for Care and Use of Laboratory Animals. The procedures were specifically approved by the Committee on the Ethics of Animal Experiments of the Fourth Military Medical University. All surgery was performed under sodium pentobarbital anaesthesia and all efforts were made to minimize suffering.

### Serum chemistry assay for bone metabolism

Serum calcium (S-Ca), phosphorus (S-P) and alkaline phosphatase (ALP) concentrations were measured by commercial kits (all from Zhongshengbeikong Biotechnology Co.) and analysed by an Automatic Biotechnology Analyser. Serum osteocalcin (OC) level was determined with radio-immunity kit (Beijing Huaying Biotechnology Research Institute) by an Automatic Radio-immunity Counter (Industrial Company of University of Science and Technology of China). Serum tartrate-resistant acid phosphatase (TRACP) and C-telopeptide of type I collagen (CTX-I) levels were determined with ELISA kits (Elabscience Biotechnology).

### Bone microarchitecture assessment by micro-computed tomography

Bone microarchitecture in left distal femur was scanned by eXplore Locus SP Pre-Clinical Specimen micro-computed tomography (micro-CT; GE Healthcare). The reconstruction and 3D quantitative analyses were performed by the desktop micro-CT system. The scanning regions were confined to the distal metaphysis, extending 2.0 mm proximally from the proximal tip of the primary spongiosa for the cancellous portion and 12.0 mm proximally from the centre of fossa intercondylica for the cortical portion (Figure 1). The 3D indices analysed in the defined region of interest (ROI) were bone mineral content (BMC), bone mineral density (BMD), relative bone volume (BV) over total volume (TV; BV/TV, %), trabecular thickness (Tb.Th.), trabecular separation (Tb.Sp.) and connectivity density (CD) of the cancellous bones; as well as BMC, BMD, mean thickness (MT), cortical area (CA), inner perimeter (IP) and outer perimeter (OP) for the cortical bones. The operator conducting the scan analysis was blinded to the treatments associated with the specimens.

**Figure 1:**
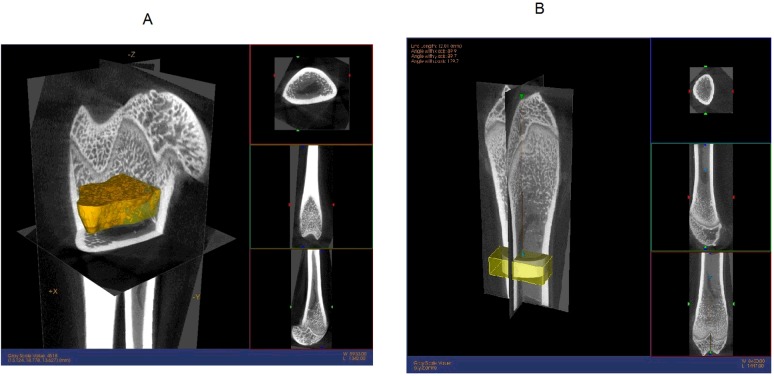
ROI in micro-CT (**A**) ROI of cancellous portion of the femur; (**B**) ROI of cortical portion of the femur.

### Measurements of bone biomechanical strength

The mechanical properties of the right femurs were determined by a three-point bending test. The biomechanical quality of the left femoral diaphysis was determined by using a CMT4204 material testing machine (Shenzhen Skyan Power Equipment Co. Ltd.) at a speed of 2 mm/min. Briefly, the left femurs were thawed at room temperature for 1 h and then placed in the material test machine with two support points separated by a distance of 20 mm. The max load, fracture energy, max stress and elastic modulus were obtained and the bone load-displacement curves were plotted simultaneously with the software. The whole femur length was measured by a Vernier caliper before machine testing. The inner/outer long/short diameters at fracture of bone were measured by the same Vernier caliper after machine testing.

### Statistical analysis

Results were expressed as the mean ± S.D. Data were evaluated using one-way ANOVA for *post-hoc* comparisons (SPSS 19.0). The data that passed the homogeneity test were analysed by the one-way ANOVA least significant difference (LSD) test. In all cases, *P<*0.05 was considered statistically significant.

## RESULTS

### Combination therapy prevented the elevation of bone turnover rate in OVX rats

S-Ca and S-P levels were obtained to reflect the bone turnover rate. S-Ca and S-P levels of rats in OVX group appeared lower compared with those in the sham group; treatment with E2 (35 μg/kg) or E2 combining G15 (160 μg/kg) seemed to have a reversing trend to the decreased S-Ca and S-P levels in OVX rats; however, these trends of differences were not significant among three groups (Table 1). Serum ALP level is an early phase phenotypic marker of osteoblastic differentiation and bone formation during the matrix maturation phase [8]. OC, one of the major non-collagenous proteins synthesized by osteoblast, which primarily deposits in the extracellular matrix of bone, is a late phase differentiation marker of osteoblast [9]. Serum TRACP and CTX-I are good indicators of osteoclasts functions and the status of bone damage [10], especially the latter one. Significant change in CTX-I levels was found before and after the treatment of osteoporosis with little the long-term variations. Thus, due to its satisfactory sensitivity and stability; it is a preferred index to evaluate the therapeutic effect [11]. In our study, Serum ALP, OC, TRACP and CTX-I concentrations were notably increased 10 weeks after OVX, suggesting a higher bone turnover rate. Treatment with E2 (35 μg/kg) or E2 combining G15 (160 μg/kg) prevented these alterations in the OVX rats (Table 1). In addition, it seemed that the preventive effects in bone turnover rate between E2 and E2 combining G15 were almost equal.

**Table 1:** Effects of combination therapy of E2 with G15 on biochemical parameters in the serum of OVX rats

	Sham (*n*=8)	OVX (*n*=8)	E2 (*n*=8)	E2 + G15 (*n*=8)
Ca (mmol/l)	2.81±0.22	2.60±0.23	2.78±0.19	2.79±0.24
P (mmol/l)	1.76±0.17	1.70±0.21	1.75±0.15	1.74±0.16
ALP (unit/l)	124±16	246±21	144±16*	134±19*
OC (ng/ml)	4.38±0.23	4.82±0.28	4.55±0.18*	4.49±0.20*
TRACP (pg/l)	2808±123	3243±132	2950±135*	2869±123*
CTX-(ng/ml)	432±22	587±20	492±19*	464±20*#

Sham/OVX: olive oil, 1.5 ml/kg, ip, three times/week; E2: 35 μg/kg, ip, three times/week; G15: 160 μg/kg, ip, three times/week; all for 6 weeks. *P<0.05 compared with OVX; ^#^*P*<0.05 compared with E2.

### Combination therapy improved BMC, BMD and bone structure in OVX rats

#### Cancellous bone

In OVX rats, BMC, BMD and BV/TV of the cancellous portion of the left femur were found to be significantly lower than the sham group. Treatment with E2 (35 μg/kg) or E2 combining G15 (160 μg/kg) reversed these changes, i.e. the treatment improved the BMC, BMD and BV/TV (Table 2). Further 3D images of femur metaphysis showed the differences in trabecular architecture among the various groups (Figures 2 and 3). Micro-CT analysis revealed lower Tb.Th. and CD, as well as higher Tb.Sp. in OVX rats as compared with that of the shams. In contrast, less OVX-induced deterioration of microstructure in trabecular was seen in E2 (35 μg/kg) or E2 combining G15 (160 μg/kg) treatment group, as shown by the increases in trabecular Tb.Th. and CD, as well as decreases in Tb.Sp. in OVX rats (Table 2). Besides, the therapeutic effect of E2 and G15 in combination was found much better than E2 alone.

**Table 2:** Micro-CT analysis of cancellous bone of the femur

	Sham (*n*=8)	OVX (*n*=8)	E2 (*n*=8)	E2 + G15 (*n*=8)
BMC (mg)	6.24±0.38	2.79±0.21	5.01±0.27*	5.54±0.26*#
BMD (mg/mL)	579±40	395±25	506±28*	552±23*#
BV/TV	0.65±0.05	0.31±0.04	0.45±0.03*	0.57±0.05*#
Tb.Th. (mm)	0.110±0.011	0.065±0.007	0.088±0.008*	0.104±0.009*#
Tb.Sp. (mm)	0.065±0.007	0.161±0.012	0.105±0.010*	0.91±0.008*#
CD (1/mm^3^)	85.13±6.20	73.69±5.73	80.44±6.00*	82.80±5.53*

Sham/OVX: olive oil, 1.5 ml/kg, ip, three times/week; E2: 35 μg/kg, ip, three times/week; G15: 160 μg/kg, ip, three times/week; all for 6 weeks. *P<0.05 compared with OVX; ^#^*P*<0.05 compared with E2.

**Figure 2:**
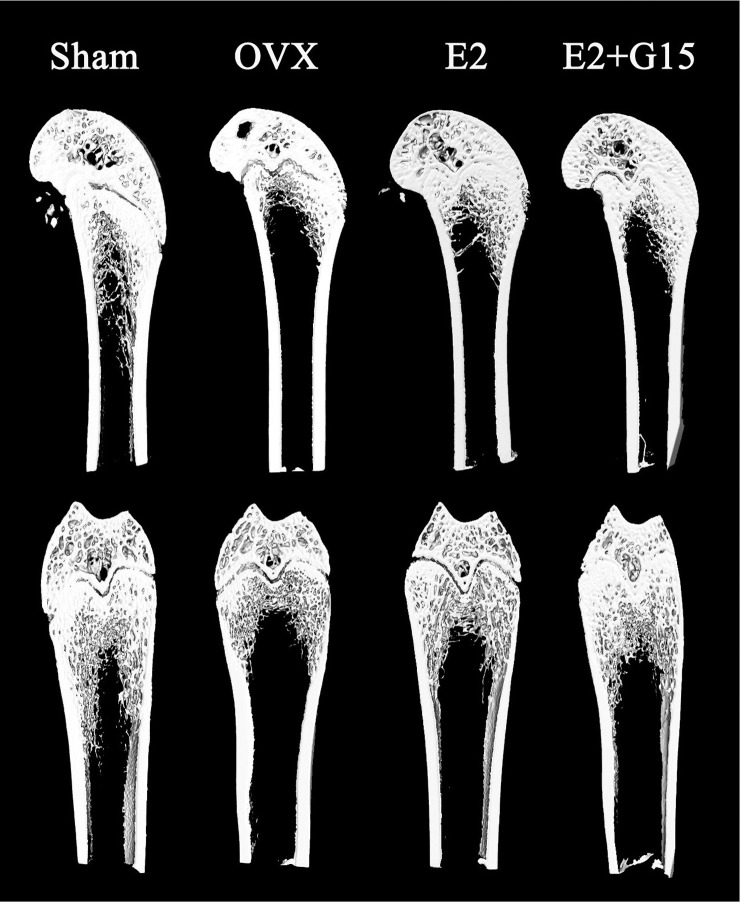
Sagittal and coronal plane of femur in different groups Sham/OVX: olive oil, 1.5 ml/kg, ip, three times/week; E2: 35 μg/kg, ip, three times/week; G15: 160 μg/kg, ip, three times/week; all for 6 weeks.

**Figure 3:**
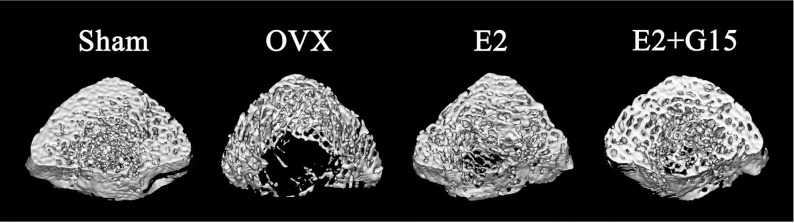
Cancellous portion of the femur in different groups Sham/OVX: olive oil, 1.5 ml/kg, ip, three times/week; E2: 35 μg/kg, ip, three times/week; G15: 160 μg/kg, ip, three times/week; all for 6 weeks.

#### Cortical bone

In OVX rats, BMC and BMD of cortical portion of the left femur were lower than those in sham group. Treatment with E2 (35 μg/kg) or E2 combining G15 (160 μg/kg) reversed those trends as BMC and BMD were found to be increased (Table 3). 3D images of the femoral metaphysis showed the differences in cortical bone architecture among all groups (Figure 4). Micro-CT analysis revealed that MT, CA, IP and OP were decreased in OVX rats as compared with sham. E2 (35 μg/kg) alone or combination therapy with G15 (160 μg/kg) prevented the OVX-induced deterioration of microstructure in cortical bone, as proved by the increases of MT, CA, IP and OP in OVX rats (Table 3). Besides, the therapeutic effects of E2 alone and E2 combining with G15 were similar.

**Table 3:** Micro-CT analysis of cortical bone of the femur

	Sham (*n*=8)	OVX (*n*=8)	E2 (*n*=8)	E2 + G15 (*n*=8)
BMC (mg)	0.181±0.005	0.164±0.007	0.180±0.005*	0.183±0.006*
BMD (mg/ml)	1138±26	1091±19	1115±21	1127±24*
MT (mm)	0.565±0.016	0.553±0.021	0.564±0.015	0.567±0.012
CA (mm^2^)	5.561±0.148	5.187±0.124	5.455±0.126*	5.503±0.135*
IP (mm)	8.007±0.152	7.659±0.136	7.901±0.180*	8.036±0.125*
OP (mm)	11.577±0.137	11.131±0.275	11.442±0.167*	11.524±0.140*

Sham/OVX: olive oil, 1.5 ml/kg, ip, three times/week; E2: 35 μg/kg, ip, three times/week; G15: 160 μg/kg, ip, three times/week; all for 6 weeks. *P<0.05 compared with OVX.

**Figure 4:**
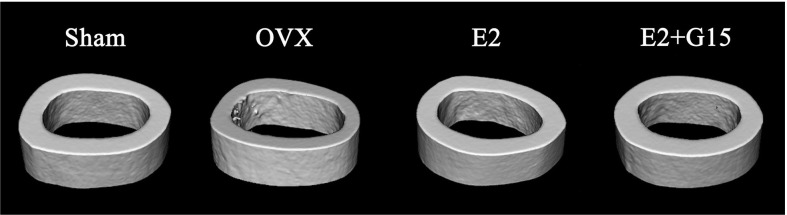
Cortical portion of the femur in different groups Sham/OVX: olive oil, 1.5 ml/kg, ip, three times/week; E2: 35 μg/kg, ip, three times/week; G15: 160 μg/kg, ip, three times/week; all for 6 weeks.

### E2 combining G15 improves biomechanical properties of bone

Three-point bending test was performed in the right femur to determine the bone strength. Extrinsic biomechanical property of the bone was evaluated by max load, fracture energy, max stress and elastic modulus. In addition, all of the femur length was measured with a Vernier caliper before the evaluation of bone biomechanical properties. As we all know, max load and fracture energy are the two indices reflecting the exterior biomechanical properties which are closely related to length, caliber and shape of the bone; meanwhile, max stress and elastic modulus are the two indices reflecting the immanent biomechanical properties which have nothing to do with their exterior characters. As shown in Table 4, OVX induced a significant decrease in max load, fracture energy, max stress and elastic modulus as compared with sham group. E2 (35 μg/kg) or combined E2 and G15 (160 μg/kg) administration significantly prevented the decrease in those indices after induction with OVX. Moreover, the reserving effects of the combination therapy on the two immanent indices, max stress and elastic modulus, were more prominent compared with those of low dose of E2 alone. Therefore, low dose of E2 combined with G15 may be a more effective therapy than E2 alone to reserve biomechanical properties of the bone in OVX rats.

**Table 4:** Effects of E2 combining G15 on biomechanical properties of bone in OVX rats

	Sham (*n*=8)	OVX (*n*=8)	E2 (*n*=8)	E2 + G15 (*n*=8)
Max load (N)	122±6	81±8	94±6*	105±5*#
Fracture energy (N·mm)	611±30	406±29	471±31*	507±25*#
Max stress (MPa)	189±10	128±9	149±9*	171±8*#
Elasticity modulus (MPa)	6368±484	4434±408	5014±417*	5691±435*#
Femur length (mm)	35.99±0.45	35.50±0.39	35.55±0.32	35.63±0.34

Sham/OVX: olive oil, 1.5 ml/kg, ip, three times/week; E2: 35 μg/kg, ip, three times/week; G15: 160 μg/kg, ip, three times/week; all for 6 weeks. *P<0.05 compared with OVX; ^#^*P*<0.05 compared with E2.

### G15 has no impact on body weight or uterus in OVX rats

The body weights of OVX model rats were markedly increased after the operation compared with those of the sham group. Treatment with low dose of E2 or E2 combining G15 could prevent the increase in body weight (Figure 5). As expected, the uterine weight of OVX model rats was significantly decreased, indicating successful surgical procedures during modelling. Treatment with E2 or E2 combining G15 could prevent the decrease in uterine weight. The addition of G15 did not further increase the uterine weight or endometrium proliferation compared with E2 administration alone (Figures 6 and 7).

**Figure 5:**
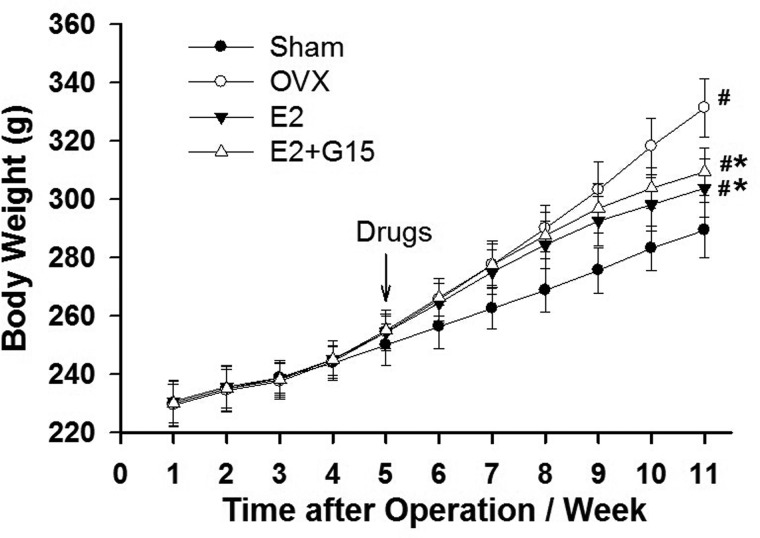
Body weight changes of rats in different groups Sham/OVX: olive oil, 1.5 ml/kg, ip, three times/week; E2: 35 μg/kg, ip, three times/week; G15: 160 μg/kg, ip, three times/week; all for 6 weeks. ^#^*P*<0.05 compared with Sham; *P<0.05 compared with OVX.

**Figure 6:**
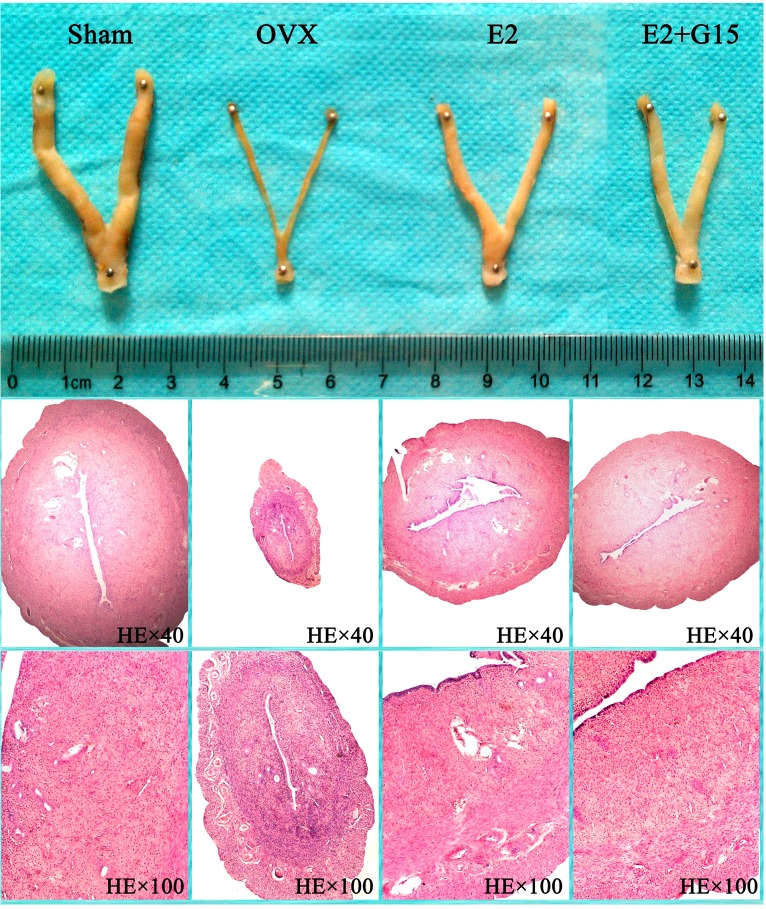
Uterus of rats in different groups Sham/OVX: olive oil, 1.5 ml/kg, ip, three times/week; E2: 35 μg/kg, ip, three times/week; G15: 160 μg/kg, ip, three times/week; all for 6 weeks.

**Figure 7:**
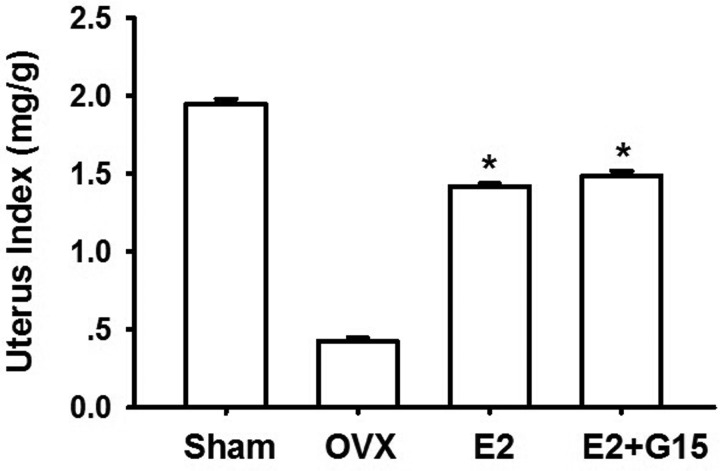
Uterus index of rats in different groups Sham/OVX: olive oil, 1.5 ml/kg, ip, three times/week; E2: 35 μg/kg, ip, three times/week; G15: 160 μg/kg, ip, three times/week; all for 6 weeks. *P<0.05 compared with OVX.

## DISCUSSION

In the present study, we used OVX-induced osteoporotic rat models to evaluate the osteoprotective effect of GPR30 and found that the combination of low-dose E2 (35 μg/kg, ip, three times/week; 6 weeks) plus G15 (160 μg/kg, ip, three times/week; 6 weeks) was effective to ameliorate the calcium loss of the bone, maintain the skeleton structure and ultimately improve the biomechanical properties. In addition, the improvements of BMC, BMD and bone structure in cancellous bone with E2 combining G15 were much higher than those of low-dose E2 alone, demonstrated by the improvements of the two immanent biomechanical parameters indices of bones, max stress and elastic modulus and proved that E2 combining G15 had better effect than that of low dose E2 alone. Besides, we found that the addition of G15 had neutralized the harmful effects on body weight or uterine endometrium. It is worth note that the results of the present study are not completely consistent to some other studies, for instance, Windahl et al. [6] found that GPR30 was not required for normal oestrogenic responses on several major well-known oestrogen-regulated effects, including increasing the BMD trabecular bones. Dennis et al. [12] found that E2 was harmful to the uterus of OVX C57B16 mice and that G15 could reverse the proliferation of uterine epithelia in a dose-related manner. As we believe, these differences may be partly attributed to different animal species, different doses of drugs, different routes of administration and different dosing frequency.

### The rationale of using combination therapy and the dosage in animals

Wronski et al. [13] found that daily 17β-E2 (molecular mass 272.38 g/mol) at 10 μg/kg subcutaneously was the lowest effective dose to protect against osteopenia in OVX SD rats (thus ∼260 nmol/kg 17β-E2 weekly) in 1988. Accordingly, we used E2 benzoate (molecular mass 376.49 g/mol) at 35 μg/kg diluted with olive oil, ip, three times per week in OVX SD rats (thus ∼280 nmol/kg E2 benzoate weekly). Considering that G15 could inhibit E2-mediated function in a dose-related manner, from 3-fold to 10-fold molar excess [12], we used 160 μg/kg of G15 (molecular mass 370.2 g/mol, thus ∼1300 nmol/kg G15 weekly) in animals, which was approximately 5-fold molar excess to E2.

### The implication of the present study on clinical prevention and treatment of post-menopausal osteoporosis

Firstly, it is important to distinguish the unique effect of GPR30 from the two classical ERs (ERα/β) on bone metabolism. As we all know, ERα and ERβ are two nuclear receptors with a wide distribution throughout the body, such as the musculoskeletal and the reproductive system. When binding to oestrogen with high affinity, the receptors activate the downstream signalling pathways and promote the transcription of various genes which may induce specific responses, including increasing BMC, BMD and promoting proliferation of the uterine endometrium. And interestingly, it seems that the functions of ERα and ERβ are not exactly the same. Lindberg et al. [14] found that some effects of oestrogen, such as increasing trabecular BMD and uterine weight, were mainly ERα mediated, whereas ERβ played only an ancillary role; ERβ, in the presence of ERα, is generally not required for or even inhibits ERα-mediated gene transcription in bone, whereas in the absence of ERα, it can partially replace the function of ERα, indicating a ‘Ying Yang’ relationship between the two ERs [15]. Meanwhile, Sims et al. [16] found that both receptor isoforms influenced bone remodelling and bone mass in a compensative way in females. Thus, they thought that ERβ may played a dual role: as a competitor of ERα at normal or low E2 levels or as an alternative for ERα, inhibiting bone turnover and preventing bone loss in the presence of high E2 levels in female mice [16]. They also concluded that both ER sub-types could influence bone turnover and trabecular structure in females, with ERα as the major effector while ERβ could only play a minor protective role [17]. Therefore, taking the above results along with our study, we hypothesize that GPR30 may be an important competitor or even a suppressor of ERα/β in terms of the bone protective effect at normal or higher oestrogen levels. Which means, when the oestrogen level is normal or higher (either with intact ovary or OVX/post-menopausal rats with appropriate amount of exogenous oestrogen), activation of GPR30 is harmful whereas suppression of GPR30 is beneficial to the bone protective role initiated by ERα/β through the ‘disinhibition’ effect, including maintaining bone mass and enhancing bone biomechanical properties. It should be noted that this finding is somewhat different that of the study by Windahl et al. [6], in which they found that the oestrogenic responses in bone did not significantly differ between WT and GPR30 KO mice for any of the tested E2 doses [6]. One of the explanations for the different, as we believe, should be that the suppressing effect of G15, the specific GPR30 antagonist, is not identical as gene KO of GPR30.

In summary, GPR30 may be able to exert its bone protective effect by inhibiting GPR30 in ERT in OVX rats, including improving parameters like BMC, BMD and trabecular structure and ultimately strengthening the biomechanical properties, especially the intrinsic biomechanical properties of the bone, including max stress and elastic modulus. At the same time, the G15 in combination could play a protective role in body weight gain or endometrial proliferation during oestrogen therapy.

Therefore, even though its role as an ER remains controversial at present [18], GPR30 is essential in bone metabolism in female patients, especially in postmenopausal women. Although some studies suggested that GPR30 might promote the development of some reproductive system malignancies [19–21], combination therapy that contains low-dose E2 and the specific antagonist G15 of GPR30 for patients with postmenopausal osteoporosis may be a good choice for clinicians.

## Abbreviations

ALP: alkaline phosphatase
BMC: bone mineral content
BMD: bone mineral density
CA: cortical area
CD: connectivity density
CT: computed tomography
CTX-I: C-telopeptide of type I collagen
E2: oestradiol
ER: oestrogen receptor
ERT: oestrogen replacement therapy
GPR30: G protein-coupled oestrogen receptor 30
IP: inner perimeter
ip: intraperitoneally
KO: knockout
MT: mean thickness
OC: osteocalcin
OP: outer perimeter
OVX: ovariectomy
ROI: region of interest
S-Ca: serum calcium
SD: Sprague—Dawley
S-P: serum phosphorus
Tb.Sp.: trabecular separation
Tb.Th.: trabecular thickness
TRACP: tartrate-resistant acid phosphatase
WT: wild-type
